# Microsporidial stromal keratitis in a cat

**DOI:** 10.1016/j.mmcr.2020.01.004

**Published:** 2020-01-16

**Authors:** Kelly A. Caruso, Seth Koch, Benjamin D. Reynolds, Kathy Szabo, Mark Mense, Ann Cali, Cameron J. Whittaker, Jeffrey Smith

**Affiliations:** a Eye Clinic for Animals, 57-63 Herbert St, Artarmon, Sydney, 2064, Australia; b The Animal Eye Clinics, 2302 Hickory Rd, Plymouth Meeting, PA, 19462, USA; c The Armed Forces Institute of Pathology, 606 Stephen Sitter Ave, Silver Spring, MD, 20910, USA; d Rutgers University Department of Biological Science, Piscatway, NJ, 08854, USA

**Keywords:** Microsporidial, Fungal, Keratitis, Feline, Veterinary

## Abstract

A 12 year-old female spayed felid presented after a 35 day history of right eye pain. On examination, a sub-epithelial opacity was identified in the cornea. A lamellar keratectomy was performed and histopathological analysis revealed low numbers of 2x4um, Gram, Hamatoxylin-eosin and Gomori methanamine-silver positive spores. Transmission electron microscopy found ultrastructural findings consistent with the phylum Microspora. To the author's knowledge, this is only the second case of microsporidial stromal keratitis reported in a felid.

## 1 Introduction

There are almost 1300 species of microsporidia recognised, and these have been recently re-classified as fungi in the phylum Microspora and kingdom Protista that infect both vertebrates and invertebrates [1]. Microsporidia are spore-forming, unicellular, obligate intracellular organisms sharing a unique organelle, the polar filament or polar tubule and are ubiquitous in nature and are distributed worldwide [1,2]. *Encephalitozoon cuniculi* is the classic microsporidial disease of mammals, and is the most extensively studied. Spontaneous infections with *E. cuniculi* have been documented in rabbits, rodents, ruminants, horses, domestic dogs, wild and captive foxes, domestic cats, psittacine birds, and non-human primates [3].

Corneal stromal microsporidiosis is rare in both humans and animals, but has been previously reported, with few human cases and one feline case [4,5]. The clinical manifestations of ocular microsporidiosis vary, and depend on both the genus involved and the immune status of the patient. There are two classical clinical presentations of ocular microsporidial infections: corneal stromal keratitis occurring in immunocompetent patients and an epithelial keratopathy and conjunctivitis seen in immunosuppressed patients [6]. However, the condition's phenotypic presentation can be mixed irrespective of the patient's immune status [7].

In immunocompromised people, especially the Human Immunodificiency Virus (HIV) positive and the organ recipient populations, microsporidia are recognised as opportunistic organisms [8,18]. Animal and environmental reservoirs of microsporidia as well as zoonotic potential are hypothesised, but not yet proven [9]. Treatment of human microsporidial infection with therapeutic agents is well documented; however there are relatively few reports of drug efficacy in animals. [10]^, STILES et al^ The single case of stromal keratitis in the feline reported prior to this case was thought to be due to *E. cuniculi,* and was cured with a keratectomy [5].

## 2 Case

A 12 year-old female spayed domestic short hair feline presented on day 0 with a 5-week history of ocular pain, corneal edema and moderate episcleral injection. The cat lived on the west coast of the USA for several years before living in Washington D.C where the case presented, and was kept indoors exclusively. The cat lived in an apartment building that faced the aviary of the National Zoo, approximately 50 yards away.

Topical triple antibiotic ointment (neomycin, bacitracin, polymixin B) was prescribed by the referring veterinarian, and did not improve the cat's eye clinically. The blepharospasm worsened, and the cat was referred to a center of veterinary ophthalmology for examination.

**Fig. 1:**
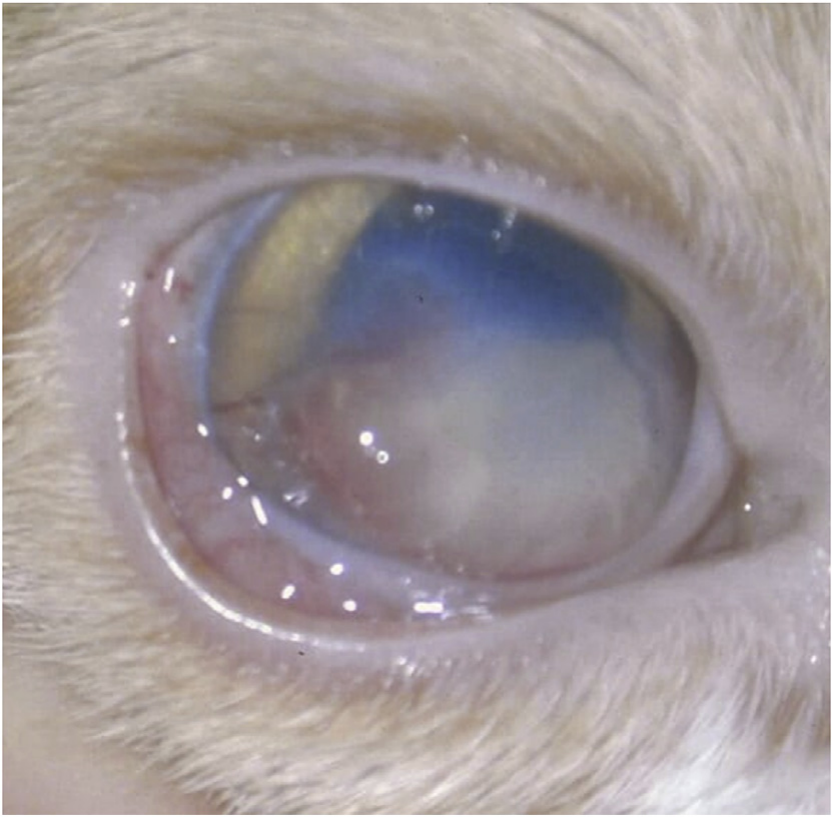
Pre-operative photograph of the corneal stromal opacity at day 0.

On examination, the cat was visual and ably navigated the examination room. The most obvious clinical sign was ocular pain of the right eye, manifested by severe blepharospasm. The cornea of the right eye was vascularised inferiorly and temporally, and the vessels extended centrally to an area of corneal sub-epithelial opacification. The corneal opacity of the right eye was yellow to white and covered the entire axial and inferior cornea, and there was moderate chemosis and hyperaemia (Fig. 1). Both pupils were responsive to light directly and consensually, and the menace response was intact in both eyes. Anterior segment exam by biomicroscopy^1^ revealed a +2 aqueous flare of the right eye, and the left eye was normal. The fundus was normal in both eyes by indirect ophthalmoscopy.^2^ The Schirmer^3^ tear test (STT) results were measured as >15mm in a minute bilaterally. Intra-ocular pressures were measured via applanation tonometry,^4^ and were 6 mmHg in the right eye and 12 mmHg in the left eye. The lower intraocular pressure of the right eye was attributed to uveitis [1]. Fluorescein tests bilaterally were negative.^5^

Corneal opacification may be due to a variety of causes, the most likely, considering the history and clinical appearance in this case would be infectious, specifically bacterial, fungal, parasitic or viral. A feline comprehensive blood screen was performed and included a complete blood count, serum chemistry, thyroid evaluation, and serologic tests for feline leukaemia virus (FeLV) and feline immunodeficiency virus antibody (FIV). Feline herpesvirus (FHV-1) tests were not performed because of poor sensitivity, and poor positive predictive value of available tests [2]. The red blood cell count (10.58 10^6^/μL; reference range 5.28–9.97 10^6^/μL) and hematocrit (52.4%; reference range 25.8–48.1%) were mildly elevated, and the eosinophil count was elevated with a percentage twice the normal value (12%; reference range 0–6%). Serum chemistry revealed elevated amylase levels (2440 U/L; reference range 600–1600U/L), elevated alkaline phosphatase levels (87 IU/L; reference range 10–42 IU/L), elevated triglycerides (320 mg/dL; reference range 30–90 mg/dL), and mildly elevated globulins (5.4g/dL; reference range 2.0–5.0 g/dL). Feline immunodeficiency virus antibody, and feline leukaemia virus antigen were not detected by ELISA tests. Urine was not available for analysis.

**Fig. 2:**
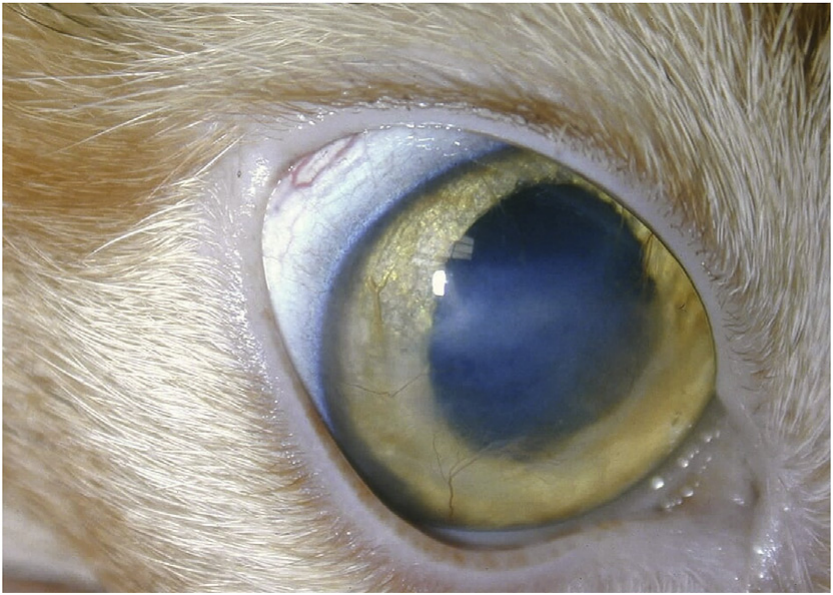
Photograph of the patient's cornea post-operatively at day 120.

Medical and surgical interventions were discussed. The goals were to relieve pain and obtain a definitive diagnosis and resolution. Considering the corneal opacity was sub-epithelial and the duration of the disease was five weeks with the progression of clinical signs worsening; surgery was recommended. A lamellar keratectomy was performed in the right eye on day 0. A bacterial culture and sensitivity was submitted prior to preparation of the cornea for surgery, and results received on day 7 revealed no growth of any bacteria. Pre-medication was a 0.3mg/kg butorphanol,^6^ 0.02mg/kg acepromazine^7^ and 0.02mg/kg atropine^8^ combination IM, Propofol^9^ was given IV to effect for induction, and the patient was maintained on isoflurane^10^ via an endotracheal tube. The cornea was prepared for surgery using triclosan antiseptic.^11^ The excised corneal sample was submitted for histopathologic analysis in 10% formalin.^12^ The cat was sent home on topical erythromycin every 8 hours and atropine ointment every 24 hours to prevent infection and relieve reflex ciliary body spasm, respectively. The keratectomy bed healed routinely with minimal vascularisation and fibrosis observed on day 120 (Fig. 2).

**Fig. 3:**
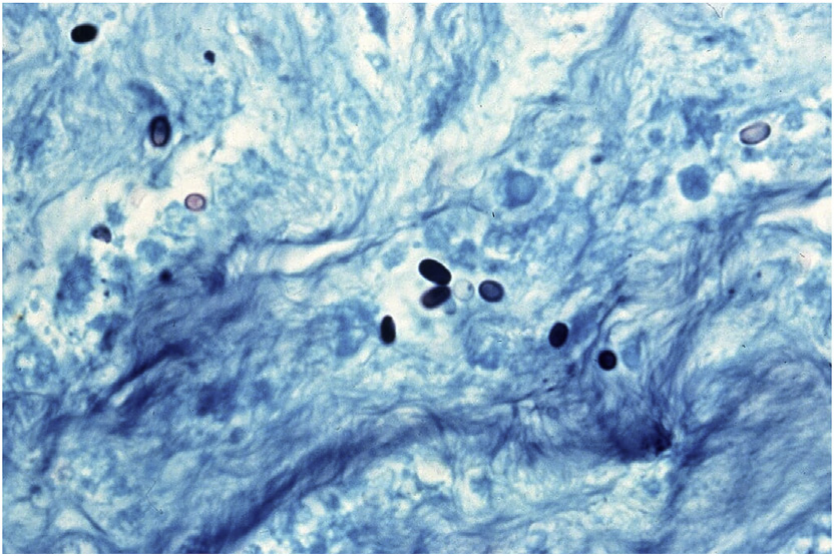
Light microscopy photomicrograph of Gomori methenamine-silver (GMS) stained cornea showing spores within the areas of corneal inflammation.

**Fig. 4:**
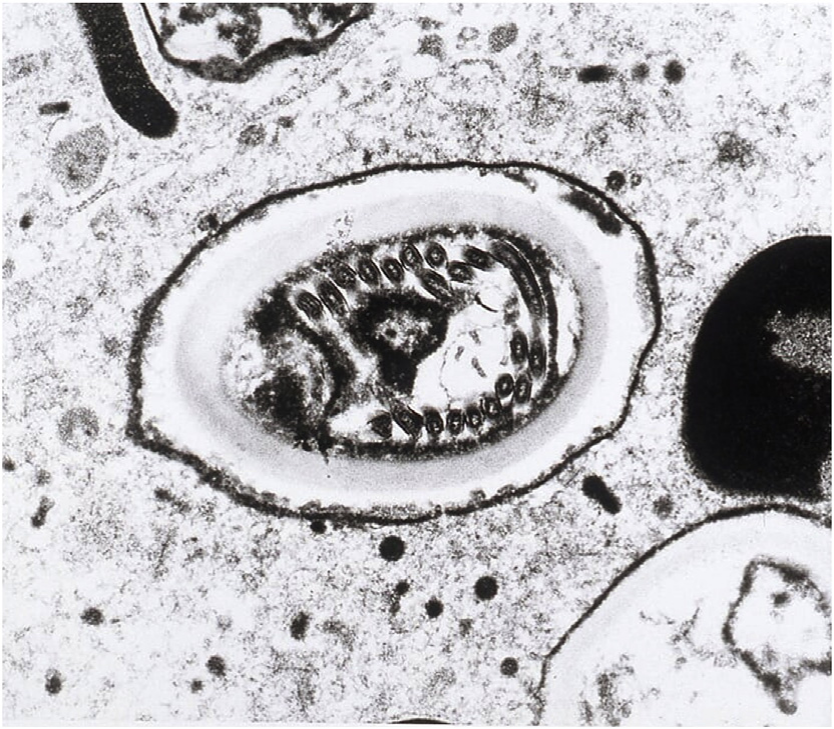
Transmission electron microscopy (TEM) of a phagocytic cell containing multiple spores at different stages in direct contact with the host cell cytoplasm.

**Fig. 5:**
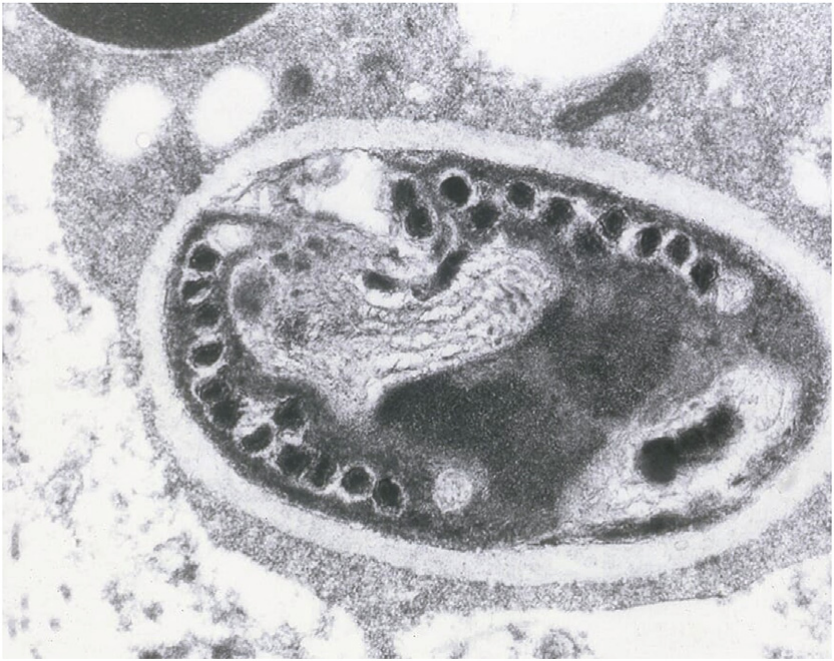
Transmission electron microscopy (TEM) of a spore section containing diplokaryotic nuclei with sections of approximately 11 polar filament coils; features consistent with the family Nosematidae and genus *Nosema*.

The definitive diagnosis of microsporidial keratitis was made histologically. Light microscopy revealed multi-focal corneal epithelial hyperplasia, intra-epithelial pustules, transmigration of neutrophils across the corneal epithelium, vascularisation and collagenolysis of the corneal stroma, and corneal edema. Within the areas of corneal inflammation, there were low numbers of 2x4um, Gram, Hamatoxylin-eosin (HE) and Gomori methanamine-silver (GMS) positive, oval protistan spores (Fig. 3). Transmission electron microscopy (TEM) showed mature spores containing sporoplasm with a single nucleus, a polar tubule with nine to eleven coils, a thin electron-dense exposure, an inner, thicker, electron-lucent endospore, a unit membrane, an anchoring disc at the anterior pole, and an electron-lucent posterior vacuole. These ultrastructural findings were consistent with the phylum Microspora, class typical of Nosematidae. Within the family Nosematidae, this organism had features most suggestive of the genus *Nosema*, and contained diplokaryotic nuclei and approximately 11 polar filament coils (Fig. 4 and Fig. 5).

## 3 Discussion

All microsporidia have an obligate intracellular life cycle, existing as environmentally resistant spores outside the host, which is their infectious stage [1]. The life cycle is postulated to be simple and direct, and most infections are acquired through ingestion or inhalation, transplacentally, or from trauma to the epithelium [2]. Transmission of microsporidia is believed to occur primarily by fecal-oral or urinary-oral routes.^1^Waterborne transmission and direct ocular contact with contaminated soil have also previously been documented as modes of transmission [11,12].

Extrusion of the polar filament from the spore, and injection of the infectious sporoplasm into the host cell is postulated to be the primary mechanism by which all microsporidia establish intracellular infection [1]. However, phagocytosis of spores by host cells may be an alternate mechanism of infection [13]. The organism then divides by a process of merogony, followed by differentiation into spores by a process called sporogony [1].

Microsporidia are recognised as opportunistic pathogens of immunocompromised people, especially the Human Immunodificiency Virus (HIV) positive, and the organ recipient populations [8,18]. Animal and environmental reservoirs of microsporidia as well as zoonotic potential are hypothesised, but not proven [1]. Treatment of human microsporidial infection with therapeutic agents is well documented; however there are relatively few reports of drug efficacy in animals [10].

Corneal stromal microsporidiosis is rare in humans, with few human cases of microsporidial stromal keratitis reported previously [4]. The ophthalmic manifestations of ocular microsporidiosis exhibit characteristic clinical features depending on the genus involved, and the immune status of the patient. There are two clinical presentations of ocular microsporidial infections: corneal stromal keratitis occurring in immunocompetent patients and an epithelial keratopathy and conjunctivitis seen in immunosuppressed patients, or mixed irrespective of the patient's immune status [4,6,7]. Stromal keratitis has previously been reported in a single feline patient prior to this case, and was thought to be due to *E. cuniculi,* and was cured with a keratectomy [5]. The organisms were present throughout the corneal stroma of the cat; however TEM to speciate the organism was not performed. If infection is limited to the corneal epithelium and conjunctiva, producing a diffuse punctate epithelial keratoconjunctivitis, the genus *Encephalitozoon* is likely; whereas with the genus *Nosema* and *Microsporidium*, the infection typically involves the stroma and keratocytes [4,6].

Diagnosis of microsporidial stromal keratitis can not easily be made by culture as microsporidia grow on tissue culture [15]. Diagnosis is made by cytologic or histopathological tissue examination, with histopathology being preferred with up to 92.3% sensitivity in the human literature [15]. TEM can be utilised for diagnosing microsporidiosis and does so by observing the polar tubule, which is a unique structure found only in microsporidia [1]. This case shows the ultrastructural features such as the number of coils in the polar tubule and arrangement of the nuclei, which can further speciate the genus of microsporidia [2]. While morphologic studies are able to distinguish between genera of microsporidia, TEM may not always distinguish species within a genus. Histochemical methods for detecting microsporidial spores are commonly used in clinical diagnostic laboratories. Microsporidia stain poorly with hematoxylin-eosin, but the Brown Brenn gram stain, and Warthrin-Starry silver can be used for detection of the fungus in tissue samples [8,16]. Serology, utilizing enzyme-linked immunosorbent assay (ELISA), may be used for screening purposes, although immunodeficient animals will not mount a reliable antibody response [8]. Polymerase chain reaction (PCR) tests have been developed and validated for multiple microsporidial species, and are now considered the gold-standard diagnostic tool for microsporidia due to their ability to detect low-levels of pathogens and high sensitivity and specificity*.* [17] Various genetic targets have been used previously with PCR detection, with the specific targets varying with the microsporidial species examined, however often a component of the small subunit ribosomal RNA is targeted [17,18].

This case represents a unique clinical finding, as it is only the second reported case of corneal microsporidia infection in the feline. This cat was systemically healthy prior to, during, and after the removal of the diseased cornea. The cat was FeLV and FIV negative, following the pattern of immune competence and corneal stromal microsporidial keratitis in humans. The elevated eosinophil count may have been an indicator, but without clinical signs of parasitic infection or disseminated fungal disease, no therapy was instituted. Although animal to animal transmission has not been documented, we question where an indoor cat may have acquired such an infection. One hypothesis is the aviary, which faced a window this cat sat in all day long. Microsporidiosis has previously been documented in birds, and infectious spores are able to survive in the environment, but the exact modes of transmission from have not been elucidated [8,9]. It may be possible that the cat traumatized the cornea, and microsporidia being ubiquitous nearby was able to opportunistically invade the cornea. This case was cured with a lamellar keratectomy, and the cornea remains disease free at day 400 post-operatively. The mode of infection for this case still remains an enigma.

Microsporidial keratoconjunctivitis has been previously identified as an occupational hazard for veterinarians [19]. An increased risk of infection may be associated with animals who possess active microsporidial infections, however a definitive reservoir of species that are pathologic to humans has yet to be discovered [20]. This disease should be considered as a potential differential diagnosis and possible workplace hazard for veterinarians due to potential risk for zoonotic infection.

## Declaration of competing interest

The authors have no personal or financial conflicts of interest.

## Footnotes

1 Kowa SL-5 slit lamp biomicroscope, Kowa Ltd.

2 Keeler Dualite Indirect Ophthalmoscope, Keeler Instruments.

3 Alcon Schirmer tear test strips, Alcon Laboratories, Inc.

4 Tono-pen XL, Mentor O & O, Inc.

5 BioGlo, Contacare Ophthalmic Pvt. Ltd.

6 Torbugesic, Fort Dodge Animal Health.

7 Aceproject, Vet8us Animal Health.

8 Atroject S.A, Vetus Animal Health.

9 Propofol, Baxter Healthcare Corporation.

10 Attane, Minrad Inc.

11 Septisol NPD with Triclosan, Steris Corporation.

12 Tissumend, Veterinary Products Laboratory.
